# Assessment of smartphone addiction and its relationship with health-promoting lifestyle profile and intuitive eating behavior: a cross-sectional study

**DOI:** 10.3389/fpsyt.2025.1682921

**Published:** 2025-10-03

**Authors:** Zhen Ding, Qichuan Pei, Tianle Fang, Lishun Xiao, Dehui Yin, Zhiming Sun

**Affiliations:** ^1^ School of Public Health, Xuzhou Medical University, Xuzhou, China; ^2^ Office of the President, Xuzhou Medical University, Xuzhou, China

**Keywords:** smartphones, questionnaire, cross-sectional study, dietary behaviors, health-promoting lifestyle

## Abstract

**Background:**

Smartphones have become an essential part of daily life, but excessive use can lead to significant issues. This study assesses smartphone addiction among college students, examining its impact on dietary behaviors and health-promoting lifestyles. The aim is to provide a basis for targeted interventions.

**Methods:**

A cross-sectional study was conducted among 1,002 students at Xuzhou Medical University in 2025 using a convenience sampling. Data were collected via questionnaires and analyzed using SPSS 21.0. The study used the Smartphone Addiction Scale-Short Version (SAS-SV), the Health-Promoting Lifestyle Profile (HPLP-II), and the Intuitive Eating Scale-2 (IES-2). Statistical methods included normality tests, descriptive statistics, and mediation analysis.

**Results:**

Among the participants, medical students scored 34 (SAS), 74 (IES), and 134 (HPLP-II), while non-medical students scored 33.5 (SAS), 74 (IES), and 134 (HPLP-II). A negative correlation was observed between smartphone addiction and both intuitive eating (r = -0.174, *p* < 0.01) and health-promoting lifestyle (r = -0.074, *p* < 0.01). However, the effect sizes for these correlations are small, suggesting that, although statistically significant, the practical significance of these relationships may be limited. Intuitive eating mediated the relationship between smartphone addiction and health-promoting lifestyle (indirect effect = -0.1452, *p* < 0.001). Approximately 25.25% of participants reported feeling addicted to smartphones.

**Conclusion:**

Smartphones have dual impacts on students’ eating behaviors and health. Excessive use is associated with disrupted eating patterns, while moderate use is associated with healthy behaviors that support well-being. It is crucial to educate students on balancing smartphone use with healthy habits is crucial. However, it is important to note that these findings, derived from a convenience sample at a single medical university, may not be fully generalizable to all college student populations. Future research with more diverse samples is needed to confirm these relationships.

## 1 Introduction

The rapid advancement of mobile internet technology has made smartphones essential part of modern life (1). However, this convenience has also led to smartphone addiction, which is becoming a significant global public health issue (2). Meta-analysis studies have indicated that the prevalence of smartphone addiction among global and Asian medical students was 26.99% (3) and 41.93% (4) respectively. The World Health Organization defines smartphone addiction as “an uncontrollable urge to use, with the behavior causing significant negative impacts on daily life, mental state, or physical health,” especially among adolescents and students.

Smartphone addiction exerts a significant influence on dietary behaviors. Excessive smartphone use can impede meal preparation and disrupt regular eating patterns, thereby fostering unhealthy habits such as reliance on fast food reliance and binge eating (5). Additionally, food marketing on social media platforms and concerns related to body image may distort individuals’ perceptions of diet and undermine intuitive eating practices. Emotional distress is frequently observed when access to smartphones is restricted (6). Behavioral addiction is characterized by factors including salience, tolerance, mood alterations, relapse, withdrawal, and conflict (7). Empirical studies have linked excessive smartphone use to increased anxiety and depression (8), cyberbullying (9), and poor dietary habits (9) among youth. These adverse outcomes can substantially affect dietary behaviors and health-related intentions, underscoring the necessity for balanced smartphone use. Therefore, acknowledging the importance of moderation is imperative.

Proactive health intention, defined as the intrinsic motivation to engage in health-promoting behaviors, encompasses commitment to a healthy lifestyle, active seeking of health information, and awareness of health risks. This construct is fundamental to effective health promotion (10). However, smartphone addiction may undermine proactive health intention by diminishing awareness of real-world health needs and reducing engagement in proactive behaviors such as exercise and dietary management (11). Empirical evidence suggests that individuals with exhibiting high levels of smartphone dependency demonstrate significantly lower in proactive health intention scores, particularly among adolescent populations (12).

**Figure 1:**
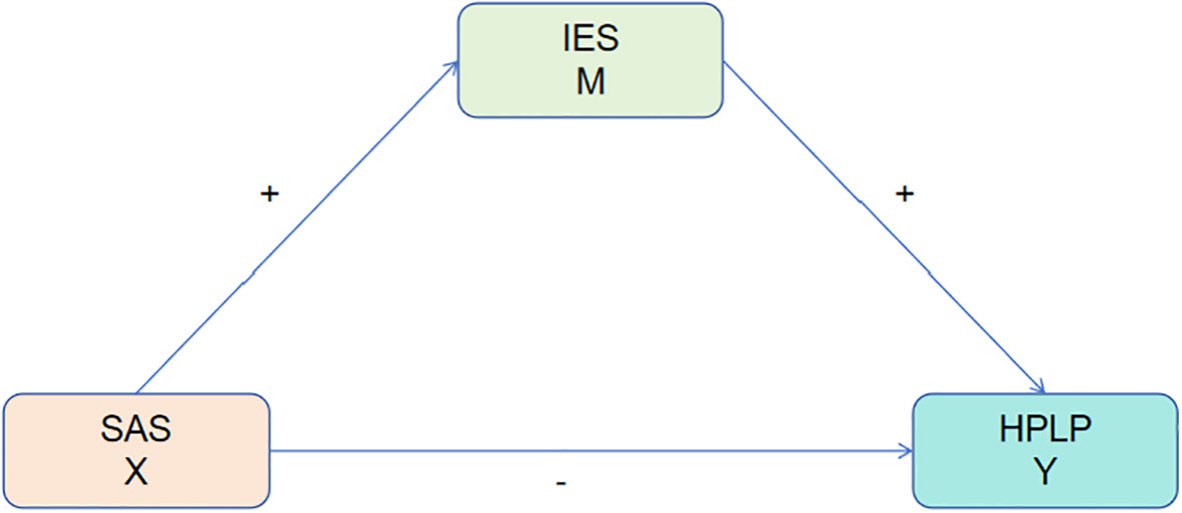
Hypothetical relationship diagram of SAS, IES, and HPLP.

Current research predominantly examines the unidirectional relationships among smartphone addiction, dietary behaviors, and health intention, frequently neglecting their dynamic interactions (13). Disrupted dietary behaviors may exacerbate emotional smartphone use, whereas strong proactive health intention may attenuate the adverse effects of addiction on eating habits (14). A comprehensive understanding of these relationships is vital essential for the development of targeted interventions aimed at students. This study seeks to analyze the interrelations among smartphone addiction, dietary behaviors, and proactive health intention, with the objective of promoting healthy adolescent development. We hypothesize a negative correlation between smartphone addiction and both dietary behaviors and health intention, and we will assess its impact on medical and non-medical students (**Figure 1**).

Hypothesis 1 posits that smartphone addiction, dietary behaviors, and health-promoting lifestyles among college students are interrelated. Specifically, smartphone addiction is positively associated with dietary behaviors and negatively associated with health-promoting lifestyles, whereas dietary behaviors exhibit a positive correlation with health-promoting lifestyles.

Hypothesis 2 posits that dietary behaviors serve as a mediating factor in the relationship between smartphone addiction and health-promoting lifestyles among college students.

## 2 Study subjects and methods

### 2.1 Sample size calculation of study subjects

The sample size was determined using the formula for cross-sectional studies:


n=Zα    2×P(1−P)/δ2

The P-value represents the prevalence of smartphone addiction among medical students in China was reported as 39.7% in a cross-sectional survey (15). Using a significance level (α) of 0.05, a corresponding Zα value of 1.96, and an allowable margin of error (δ) set at 5% in the present study, the minimum required sample size was calculated to be 368.

In February 2025, students from Xuzhou Medical University participated in this study. Of the 1,020 questionnaires distributed, 18 were excluded due to abnormal height and weight values or missing data, yielding 1,002 valid responses and a response rate of 98.24%.

### 2.2 Research methodology

A questionnaire survey was conducted to collect data on students’ personal information, mobile phone usage, dietary behaviors, and proactive health intentions.

#### 2.2.1 Smartphone addiction scale - short version

The Smartphone Addiction Scale-Short Version (SAS-SV) is a self-reported instrument comprising 10 items designed to assess smartphone addiction (16). It employs a 6-point Likert scale to measure usage over the preceding six months, with higher scores reflecting greater dependency (16). The SAS-SV has been extensively utilized and validated across diverse cultural settings. In particular, the scale has demonstrated strong psychometric properties within the Chinese population (16), and conducted a translation and validation study among Chinese college students, confirming the unidimensional factor structure of the SAS-SV and reporting excellent internal consistency (Cronbach’s α = 0.89). These findings provide robust evidence supporting the scale’s validity and reliability in the cultural context relevant to the present study. Additionally, its applicability has been corroborated by another Chinese research (17). Consistent with Kwon et al. (18), this study adopted cut-off scores for smartphone addiction of ≥33 for female students and ≥31 for male students. In the current sample, the SAS-SV demonstrated a Cronbach’s alpha of 0.878, aligning with previous Chinese validation studies and indicating high reliability.

#### 2.2.2 Health-promoting lifestyle profile

The Health-Promoting Lifestyle Profile II (HPLP-II) comprises 52 items distributed across six dimensions: Physical Activity (PA), Health Responsibility (HR), Stress Management (SM), Nutrition (NU), Interpersonal Relations (IR), and Spiritual Growth (SG) (19). Data collection involved a demographic questionnaire and the HPLP-II instrument, which evaluates health-promoting behaviors. The demographic questionnaire captured variables including age, gender, academic major, place of residence, economic status, weight, and height. This study employed the officially translated and validated Chinese version of the HPLP-II developed by Teng et al. (20). Their validation study, conducted among Taiwan adults, demonstrated satisfactory reliability and acceptable model fit indices, thereby supporting the instrument’s applicability within Chinese-speaking populations. Although originally developed in Taiwan, the shared written language and cultural similarities justify its use in Mainland China. Moreover, prior research has successfully implemented this version in mainland Chinese samples. In the current study, the HPLP-II exhibited a Cronbach’s alpha coefficient of 0.965, indicating excellent internal consistency and confirming its reliability for assessing health-promoting lifestyles among mainland Chinese university students.

#### 2.2.3 Intuitive eating scale-2

The Intuitive Eating Scale-2 (IES-2), developed by Tylka and Babbott et al. (21), consists of four factors: Unconditional Permission to Eat (6 items), Eating for Physical Rather than Emotional Reasons (8 items), Eating in Response to Hunger and Satiety Cues (6 items), and Body-Food Choice Congruence (3 items), comprising a total of 23 items. Responses are measured on a 5-point Likert scale ranging from 1 (Strongly Disagree) to 5 (Strongly Agree), with higher scores reflecting greater levels of intuitive eating. The IES-2 has been validated within the Chinese cultural context. Specifically, Ji et al. (22) examined the psychometric properties of the IES-2 in a large sample of Chinese adults and confirmed its four-factor structure through psychometric network analysis. Their results support the cross-cultural validity and applicability of the IES-2 for assessing intuitive eating in China. In the present study, the Cronbach’s alpha for the IES-2 was 0.856, consistent with the good internal consistency reported by Ji et al., thereby further substantiating its reliability for the current study population.

### 2.3 Statistical analysis

Data were analyzed using SPSS version 21.0. The Shapiro-Wilk test was employed to assess normality; non-normally distributed data were presented as medians with interquartile ranges (M [P25, P75]). The Mann-Whitney U test was used to compare two groups, while the Kruskal-Wallis H test was applied for comparisons among multiple groups. Spearman’s correlation analysis was conducted to examine relationships among variables. Continuous variables were reported as medians (P25, P75), and categorical variables as frequencies and percentages. A significance level of α = 0.05 was adopted. The Bootstrap method (Model 4) was utilized to test the mediating effect of dietary behavior on the relationship between smartphone addiction and health-promoting lifestyle, with 5,000 resamples used to calculate the 95% confidence interval (CI). A path was considered significant if the 95% CI did not include zero, and *p* < 0.05 were deemed statistically significant.

## 3 Results

### 3.1 Common method bias

The Harman single-factor test revealed the presence of 15 factors with eigenvalues exceeding 1. The first factor accounted for 25.44% of the total variance, which is below the 40% threshold, suggesting that common method bias is not a significant concern in this study.

### 3.2 Study population characteristics

This study comprised 1,002 participants, who were categorized and analyzed as either non-medical or medical students (see **Table 1**). Among the participants, 198 males (55.93%) and 381 females (58.80%) were identified as exhibiting smartphone addiction (refer to **Table 2**). No significant differences were observed with respect to gender, living expenses, only-child status, mobile phone usage, or body mass index (BMI). However, a significant difference was detected in household registration status: 455 participants (45.41%) were urban residents, whereas 547 (54.59%) were rural residents (*χ* ^2^ = 9.749, *p* = 0.002). First-year students constituted the majority at 57.19% (n = 573), followed by second-year students at 21.85% (n = 219). Additionally, a significant difference in the distribution of academic year was identified (*χ* ^2^ = 10.451, *p* = 0.015), suggesting variations in student composition across disciplines.

**Table 1:** Basic characteristics of the study population.

Variable		Non-medical students	Medical students	*χ^2^*	*P*	Total	Percentage
Gender				0.035	0.089		
	Male	183	171			354	35.33%
	Female	331	317			648	64.67%
Native place				9.749	0.002		
	Urban	258	197			455	45.41%
	Rural	256	291			547	54.59%
Only child or not				0.070	0.769		
	Yes	197	191			388	38.72%
	No	317	297			614	61.28%
Staying up late using a mobile phone				0.842	0.386		
	No	388	356			744	74.25%
	Yes	126	132			258	25.75%
Grade				10.451	0.015		
	First year	271	302			573	57.19%
	Second year	117	120			219	21.85%
	Third year	88	58			146	14.57%
	Fourth and above	38	26			64	6.39%
Living expenses				2.264	0.520		
	Less than 800 yuan	6	10			16	1.60%
	800–1600 yuan	192	178			370	36.93%
	1600–2400 yuan	266	261			527	52.59%
	More than 2400 yuan	50	39			89	8.88%
Duration of mobile phone usage				3.051	0.549		
	Less than 1 hour	9	5			14	1.40%
	1–3 hours	63	47			110	10.98%
	3–5 hours	195	198			393	39.22%
	5–8 hours	180	171			351	35.03%
	More than 8 hours	67	67			134	13.37%
BMI				3.977	0.264		
	<18.5	61	72			133	13.27%
	18.5-24	318	285			603	60.18%
	24-28	83	91			174	17.37%
	>28	52	40			92	9.18%

**Table 2:** Smartphone addiction among university students with different characteristics.

Variable		N (%)	Addiction		*χ^2^*	*P*
			Yes	No		
Total		1002(100)	579(57.78)	423(42.22)		
Gender					0.770	0.385
	Male	354(35.33)	198(55.93)	156(44.07)		
	Female	648(64.67)	381(58.80)	267(41.20)		
Grade					5.605	0.132
	First year	573(57.19)	320(55.85)	253(44.15)		
	Second year	219(21.85)	134(61.19)	85(38.81)		
	Third year	146(14.57)	93(63.70)	53(36.30)		
	Fourth and above	64(6.39)	32(50.00)	32(50.00)		
Native place					2.755	0.108
	Urban	455(45.41)	250(54.95)	205(45.05)		
	Rural	547(54.59)	329(60.15)	218(39.85)		
BMI					1.873	0.599
	≤18.5	133(13.27)	80(60.15)	53(39.85)		
	18.5-24	603(60.18)	338(56.05)	265(43.95)		
	24-28	174(17.37)	105(60.34)	69(39.66)		
	>28	92(9.18)	56(60.87)	36(39.13)		
Only child or not					16.788	<0.001
	Yes	388(38.72)	193(49.74)	195(50.26)		
	No	614(61.28)	386(62.87)	228(37.13)		
Living expenses					4.781	0.189
	Less than 800 yuan	16(1.60)	11(68.75)	5(31.25)		
	800–1600 yuan	370(36.93)	227(61.35)	143(38.65)		
	1600–2400 yuan	527(52.59)	295(55.98)	232(44.02)		
	More than 2400 yuan	89(8.88)	46(51.69)	43(48.31)		
Staying up late using a mobile phone					92.977	<0.001
	No	744(74.25)	364(48.92)	380(51.08)		
	Yes	258(25.75)	215(83.33)	43(16.67)		
Duration of mobile phone usage					94.348	<0.001
	Less than 1 hour	14(1.40)	1(7.14)	13(92.86)		
	1–3 hours	110(10.98)	36(32.73)	74(67.27)		
	3–5 hours	393(39.22)	199(50.64)	194(49.36)		
	5–8 hours	351(35.03)	233(66.38)	118(33.62)		
	More than 8 hours	134(13.37)	110(82.09)	24(17.91)		

### 3.3 Reliability analysis

The reliability analysis results are presented in the **Table 3**. The reliability coefficients for the SAS-SV, IES-2, and HPLP-II scales, as well as their secondary dimensions, ranged from 0.7 to 1.0, indicating good internal consistency and high reliability.

**Table 3:** Reliability analysis of each scale.

Variable	Cronbach’s alpha coefficient (α)	Number of items
SAS-SV	0.878	10
IES-2	0.856	23
NU	0.797	9
HR	0.920	9
SG	0.928	9
SM	0.870	8
PA	0.887	8
IR	0.911	9
HPLP-II	0.965	52

### 3.4 Differences analysis

#### 3.4.1 Analysis of differences in smartphone addiction scores

Given the non-normal distribution of the data, the median and interquartile range (M [P25, P75]) were employed for descriptive analysis (see **Table 4**). Comparative analysis of SAS-SV scores among university students indicated no significant differences between medical and non-medical students with respect to gender, living expenses, household registration, and BMI. Conversely, significant differences were observed based on academic year, late-night mobile phone use, and whether the student was an only-child status (*p* < 0.001). Students who engage in late-night mobile phone use exhibited higher addiction scores, suggesting a strong association between nocturnal usage and smartphone addiction. Furthermore, extended duration of mobile phone use was positively correlated with increased addiction scores (*p* < 0.001). Prolonged exposure to repetitive informational stimuli appears to impair self-control, with addictive behaviors becoming more pronounced after five hours of use.

**Table 4:** Smartphone addiction scores among university students with different characteristics.

Variable		Total	Percentage	SAS		Z	*P*
				Medical students	Non-medical students		
Gender						-1.72	0.085
	Male	354	35.33%	32(25, 38)	33(25, 40)		
	Female	648	64.67%	34(29, 40)	34(29, 39)		
Grade						8.625	0.035
	First year	573	57.19%	33(27, 38)	34(28, 38)		
	Second year	219	21.85%	34(28, 39.5)	35(27.75, 39)		
	Third year	146	14.57%	36(30, 40.75)	34(26, 40)		
	Fourth and above	64	6.39%	30(24, 37.75)	33.5(27.5, 40.75)		
Native place						-1.259	0.208
	Urban	455	45.41%	33(27, 39)	34(26, 39)		
	Rural	547	54.59%	34(27, 39)	34(29, 39)		
BMI						1.731	0.630
	≤18.5	133	13.27%	35(29.5, 38)	33(29, 38)		
	18.5-24	603	60.18%	33(27, 39)	34(28, 39)		
	24-28	174	17.37%	32(26, 39)	35(29, 40)		
	>28	92	9.18%	30.25(37, 41)	30.5(25.25, 37.75)		
Only child or not						-4.044	<0.001
	Yes	388	38.72%	32(25, 38)	33(26, 38)		
	No	614	61.28%	34(29, 40)	34(29, 39.5)		
Living expenses						6.420	0.093
	Less than 800 yuan	16	1.60%	35(27.75, 41.25)	37.5(33, 41.75)		
	800–1600 yuan	370	36.93%	34(28, 39)	34(29, 39.25)		
	1600–2400 yuan	527	52.59%	33(27, 39)	34(27, 39)		
	More than 2400 yuan	89	8.88%	33(22.75, 40)	32(27, 38)		
Staying up late using a mobile phone						-11.628	<0.001
	No	744	74.25%	32(26, 37)	32(25.25, 37)		
	Yes	258	25.75%	39(33, 43)	38(34, 42)		
Duration of mobile phone usage						160.952	<0.001
	Less than 1 hour	14	1.40%	10(10, 20.5)	10(10, 32.5)		
	1–3 hours	110	10.98%	27(22, 34)	29(22, 34)		
	3–5 hours	393	39.22%	32(25, 37)	32(26.75, 37)		
	5–8 hours	351	35.03%	35(31, 40)	35(30, 40)		
	More than 8 hours	134	13.37%	40(33, 47)	38(35, 44)		

#### 3.4.2 Analysis of differences in health-promoting lifestyle scores

Health-promoting lifestyle scores were presented as median (P25, P75) due to the non-normal distribution of the data (**Table 5**). Comparative analysis among university students indicated no significant differences between medical and non-medical students with respect to being an only child. However, significant differences were identified based on gender and academic year (*p* < 0.001). Scores generally decreased as students progressed through their academic years, which may be attributed to increased academic pressure, occupational stress, and social engagements. Additionally, significant differences in scores were observed across BMI categories (*p* = 0.048) and were associated with household registration status, living expenses, frequency of late-night mobile phone use, and duration of usage.

**Table 5:** Health-promoting lifestyle scores among university students with different characteristics.

Variable		Total	Percentage	HPLP-II		Z	*P*
				Medical students	Non-medical students		
Gender						-3.397	<0.001
	Male	354	35.33%	134(111, 151)	130(108, 147)		
	Female	648	64.67%	134(118, 154)	136(119.5, 152)		
Grade						15.569	<0.001
	First year	573	57.19%	138(120, 156)	134(117, 151.25)		
	Second year	219	21.85%	137(113,154.5)	135(117.25,152.25)		
	Third year	146	14.57%	127(110.25,141.25)	128.5(115, 143.25)		
	Fourth and above	64	6.39%	127(116, 151.25)	124(114, 149.25)		
Native place						-3.232	<0.001
	Urban	455	45.41%	136(118, 156)	138(120.5, 155)		
	Rural	547	54.59%	131(114.25, 150.75)	131(113, 147)		
BMI						7.915	0.048
	≤18.5	133	13.27%	130(109.5, 153.5)	134(114.5, 150.5)		
	18.5-24	603	60.18%	137(119, 155.25)	135(118, 152)		
	24-28	174	17.37%	130(117, 148)	128(114, 144)		
	>28	92	9.18%	129(109.5, 147.75)	132.5(106, 156)		
Only child or not						-1.493	0.136
	Yes	388	38.72%	134(116, 156)	136(116, 156)		
	No	614	61.28%	134(117, 151)	132(116, 147)		
Living expenses						16.736	<0.001
	Less than 800 yuan	16	1.60%	109.5(98.5, 160.5)	104.5(92.25, 146.5)		
	800–1600 yuan	370	36.93%	128.5(113, 148)	133.5(114, 150.25)		
	1600–2400 yuan	527	52.59%	136.5(119.75, 156)	133(117.5, 150.5)		
	More than 2400 yuan	89	8.88%	141.5(117.75, 157.25)	138(122, 160)		
Staying up late using a mobile phone						-3.267	<0.001
	No	744	74.25%	135(117, 155)	136(117.25, 153)		
	Yes	258	25.75%	130.5(113, 147.25)	127(110.25, 142.75)		
Duration of mobile phone usage						12.253	0.016
	Less than 1 hour	14	1.40%	148(78, 204.5)	105(52, 181)		
	1–3 hours	110	10.98%	147(123, 157)	133(102, 156)		
	3–5 hours	393	39.22%	137(117, 154)	136(120, 153)		
	5–8 hours	351	35.03%	130(117, 148.75)	133(114, 148)		
	More than 8 hours	134	13.37%	128(112, 148)	131(108, 148)		

#### 3.4.3 Analysis of differences in dietary behavior scores

Given the non-normal distribution of the data, dietary behavior scores were presented as medians with interquartile ranges (M [P25, P75]). Analysis of the IES among university students revealed no significant differences between medical and non-medical students regarding mobile phone usage, late-night usage, only-child status, living expenses, and BMI. However, significant gender differences were observed (*p* = 0.006), suggesting that gender influences dietary behaviors. Additionally, dietary behavior scores varied significantly across academic years (*p* = 0.023), potentially reflecting fluctuations associated with changes in academic pressure and lifestyle pace. Furthermore, significant differences were identified between urban and rural students (*p* = 0.03), highlighting the influence of food culture (23). Consistent with these findings, Wang et al. (24) reported that Westernized dietary patterns are more prevalent in urban areas, whereas traditional plant-based diets remain predominant in rural regions. The results are summarized in **Table 6**.

**Table 6:** Dietary behavior scores among university students with different characteristics.

Variable		Total	Percentage	IES		Z	*P*
				Medical students	Non-medical students		
Gender						-2.764	0.006
	Male	354	35.33%	72(68, 79)	73(68, 79)		
	Female	648	64.67%	74(69, 79)	74(69, 80)		
Grade						9.564	0.023
	First year	573	57.19%	73(69, 79)	73(68, 79)		
	Second year	219	21.85%	76(68, 81)	75(70, 80)		
	Third year	146	14.57%	74(69, 79)	75.5(72, 82)		
	Fourth and above	64	6.39%	75.5(70, 80.5)	74.5(68.75, 79.25)		
Native place						-2.166	0.03
	Urban	455	45.41%	74(69, 80)	75(69, 81.5)		
	Rural	547	54.59%	73(69, 79)	73(69, 78)		
BMI						2.135	0.545
	≤18.5	133	13.27%	74(68, 81)	72.5(68, 77)		
	18.5-24	603	60.18%	74(69, 79)	74(69, 80)		
	24-28	174	17.37%	75(68, 80)	74(68, 80)		
	>28	92	9.18%	72.5(68, 80)	74(69, 79.75)		
Only child or not						-1.405	0.160
	Yes	388	38.72%	74(69, 80)	74(69, 82)		
	No	614	61.28%	74(69, 78.5)	74(69, 79)		
Living expenses						5.651	0.130
	Less than 800 yuan	16	1.60%	82(72.5, 88.75)	75(63.25, 80)		
	800–1600 yuan	370	36.93%	73(68, 78)	73.5(69, 78)		
	1600–2400 yuan	527	52.59%	74(69, 80)	74(69, 80)		
	More than 2400 yuan	89	8.88%	75(67.75, 79.25)	74(69, 81)		
Staying up late using a mobile phone						-0.928	0.353
	No	744	74.25%	73.5(69, 79)	74(69, 80)		
	Yes	258	25.75%	75(68, 80)	74(69.25, 79)		
Duration of mobile phone usage						2.957	0.565
	Less than 1 hour	14	1.40%	72(26, 90)	68(23, 99.5)		
	1–3 hours	110	10.98%	74(68, 79)	76(67, 82)		
	3–5 hours	393	39.22%	73(69, 79)	74(69, 78)		
	5–8 hours	351	35.03%	74(69, 79)	74(69, 80)		
	More than 8 hours	134	13.37%	74(68, 80)	74(69, 80)		

### 3.5 Correlation analysis

Smartphone addiction scores demonstrated statistically significant yet weak negative correlations with various dimensions of a health-promoting lifestyle. For instance, the correlation with the total HPLP-II score was r = -0.074 (*p* < 0.01). These findings suggest that although a relationship exists, the practical impact of smartphone addiction on overall health-promoting lifestyle is limited. A somewhat stronger, albeit still modest, negative correlation was identified with dietary behavior (r = -0.174, *p* < 0.01). In contrast, a moderate positive correlation was observed between health-promoting lifestyle dimensions and dietary behavior (r = 0.399, *p* < 0.01). The detailed results are presented in **Table 7**.

**Table 7:** Correlation analysis of various influencing variables with smartphone addiction.

	SAS-SV	IR	NU	HR	SG	PA	SM	HPLP-II	IES-2
SAS-SV	1								
IR	-0.116**	1							
NU	-0.068*	0.496**	1						
HR	-0.119**	0.493**	0.557**	1					
SG	-0.153**	0.599**	0.566**	0.635**	1				
PA	-0.222**	0.430**	0.465**	0.561**	0.589**	1			
SM	-0.162**	0.574**	0.539**	0.582**	0.765**	0.566**	1		
HPLP-II	-0.074*	0.310**	0.344**	0.278**	0.378**	0.221**	0.426**	1	
IES-2	-0.174**	0.764**	0.742**	0.785**	0.877**	0.731**	0.831**	0.399**	1

**p < 0.01, *p < 0.05.

### 3.6 Mediation analysis

**Figure 2:**
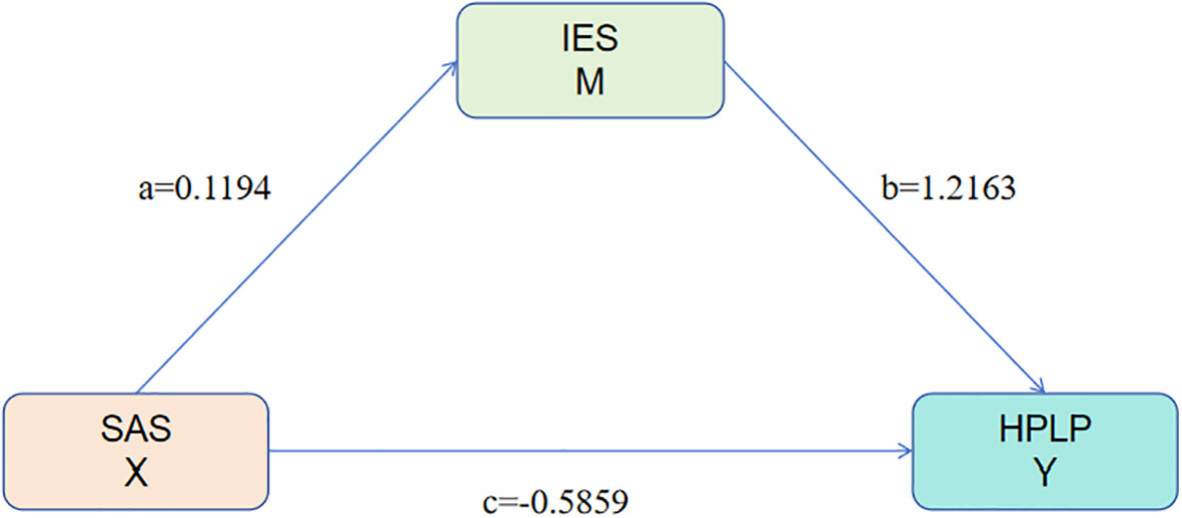
Path coefficients of SAS, HPLP, and IES.

A mediation analysis was performed using SAS as the independent variable, IES as the mediator, and HPLP as the dependent variable (see **Table 8**). The findings indicated that smartphone addiction exerts a direct negative effect on health-promoting lifestyle, with a coefficient of -0.5859. The IES variable mediates 32.95% of this effect, demonstrating its significant role in the relationship between smartphone addiction and health-promoting lifestyle. Specifically, higher levels of smartphone addiction are associated with increased dietary behavior scores (a = 0.1194) and decreased health-promoting lifestyle scores (c = -0.5859). Moreover, healthier dietary behaviors are positively correlated with higher health-promoting lifestyle scores (b = 1.2163) (**Figure 2**). These results suggest that dietary behavior serves as a mediator in the relationship between smartphone addiction and health-promoting lifestyle, thereby supporting the study hypotheses.

**Table 8:** Mediating effect of dietary behavior on the relationship between smartphone addiction and health-promoting lifestyle.

	Effect	Boot.SE	Boot 95% CI		*P*	Proportion
			Lower	Upper		
Total effect	-0.4407	0.0911	-0.6194	-0.2619	<0.001	100%
Direct effect	-0.5859	0.0806	-0.7440	-0.4277	<0.001	132.95%
Indirect effect	0.1452	0.0669	0.0199	0.2850	<0.001	32.95%

## 4 Discussion

This study investigated smartphone addiction and its association with intuitive eating and health-promoting lifestyles among university students. The primary objective was to assess addiction levels and their influence on dietary habits and health behaviors, thereby providing a foundation for targeted intervention strategies. The analysis revealed no significant differences in basic demographic characteristics -such as gender, living expenses, only-child status, mobile phone usage duration, and BMI - between non-medical and medical students. However, significant differences were observed in household registration and academic year. Given the non-normal distribution of scores across the three measurement scales, median values with interquartile ranges (M [P25, P75]) were employed for statistical analysis. No significant group differences in smartphone addiction were identified concerning gender, living expenses, household registration, or BMI; however, mobile phone usage duration differed significantly between groups. The findings suggest that interventions targeting nighttime phone use and average daily usage duration may contribute to reducing the risk of smartphone addiction among university students. Furthermore, while the within-group prevalence of smartphone addiction was similar between males (55.93%) and females (58.80%), the overall number of addicted participants was higher among females (381 out of 579 addicted individuals, 65.8%). This apparent gender skew is largely attributable to the imbalanced gender distribution in our sample, wherein female students constituted a majority (64.67%). Therefore, the higher absolute number of female addicted participants should not be interpreted as indicating a greater susceptibility to smartphone addiction among females. Instead, these findings suggest that smartphone addiction is prevalent across both genders, with no substantial difference in risk based on sex alone.

Health-promoting lifestyle scores did not differ significantly between medical and non-medical students when considering only-child status. However, significant variations were observed based on household registration, which may reflect the influence of urban versus rural living environments. Additionally, differences in scores across BMI categories suggest a potential association between BMI and engagement in health-promoting behaviors. Higher living expenses correlated with improved health-promoting lifestyle scores, implying greater access to health-enhancing resources. Furthermore, students who reported staying up late using mobile phones exhibited lower health-promoting lifestyle scores. A longer duration of mobile phone use was also linked to reduced scores, indicating that excessive mobile phone usage may detract from time allocated to health-promoting activities.

No significant differences were observed in intuitive eating behavior scores between non-medical and medical students with respect to mobile phone usage, late-night usage, only-child status, living expenses, and BMI. Correlation analysis revealed a negative association between smartphone addiction and a health-promoting lifestyle, suggesting that engagement in healthy behaviors may mitigate digital addiction. Participation in regular offline activities and effective health management strategies appear to reduce reliance on the virtual environment. Additionally, smartphone addiction was found to be negatively correlated with dietary behavior; individuals exhibiting healthier eating patterns demonstrated greater awareness of physiological cues and lower dependence on smartphones (25). Conversely, individuals with disordered eating behaviors may resort to smartphone use to compensate for psychological deficits. In our study, we observed significant differences in dietary behavior scores between urban and rural students (p = 0.03). Specifically, urban students had a median IES-2 score of 74 (IQR: 69, 80.5), while rural students had a median score of 73 (IQR: 69, 79). This suggests that urban students exhibited slightly higher levels of intuitive eating behaviors compared to their rural counterparts. Urban areas typically have greater access to a variety of food options, including a wider range of fresh produce, health food stores, and diverse dining options. This diversity may encourage healthier eating habits and a greater awareness of nutritional needs. Additionally, urban environments often have more resources for health education and promotion, which can influence dietary behaviors. In contrast, rural areas may have more limited access to fresh and diverse food options. The availability of convenience stores and fast-food outlets may be higher, leading to a greater reliance on processed and high-calorie foods. This can impact the overall quality of diet and intuitive eating practices. Most dimensions of a health-promoting lifestyle were positively correlated, underscoring their synergistic interrelations (26). Regular exercisers tended to place greater value on social connections, while those emphasizing health responsibility maintained better sleep hygiene (27). Furthermore, positive correlations between health-promoting lifestyle dimensions and dietary behavior highlight the integrative nature of health practices.

Path coefficient analysis demonstrates that smartphone addiction exerts a significant influence on dietary behavior, indicating that increased smartphone usage can modify eating patterns. Furthermore, this addiction is negatively correlated with a health-promoting lifestyle, highlighting the detrimental impact of digital dependence on healthy living. Healthy eating is linked to improved energy levels and fosters a sense of control over one’s health, thereby establishing a positive feedback loop that extends to exercise and sleep (28, 29). Additionally, dietary behavior mediates the relationship between smartphone addiction and a health-promoting lifestyle, as smartphone use is associated with distorted dietary cognition and behaviors that undermine healthy living.

An educational intervention targeting university students should emphasize literacy by teaching responsible smartphone use and addressing the effects of addiction on physical awareness and dietary habits. Mindfulness training (30) has been shown to enhance awareness of physiological signals (31). Nutrition education should incorporate principles of intuitive eating to assist students in resisting external food cues. Additionally, lifestyle education should focus on dietary behaviors and promote healthy habits, such as regular exercise and positive social interactions (32).

The campus environment exerts a significant influence on the behaviors of university students (33, 34). Higher education institutions should enhance mental health support by offering counseling services and resources for individuals affected by smartphone addiction. A collaborative framework encompassing family, school, and society is essential. Families are encouraged to promote healthy lifestyles, with parents reducing smartphone dependence and fostering regular eating habits and physical activity. Societal support also plays a critical role in encouraging healthy behaviors (35, 36). Future research should incorporate macro-level factors, such as family environment and social culture, to develop a comprehensive model of influences on students’ healthy lifestyles, thereby providing deeper insights and practical strategies for promoting well-being in contemporary society. Although the present study employed Chinese versions of scales previously validated in similar populations, future investigations should further examine the convergent and discriminant validity of these instruments specifically within the context of mainland Chinese university students to strengthen their psychometric robustness for this demographic. The findings of this study should be interpreted in light of its limitations. The use of a convenience sampling method from a single medical university restricts the generalizability of the results. Participants from Xuzhou Medical University may not be representative of the broader population of Chinese college students, who differ substantially in academic pressures, regional cultures, and socioeconomic backgrounds. Consequently, caution is advised when generalizing these findings to other student populations. Additionally, reliance on self-reported data introduces potential biases; participants may underreport undesirable behaviors, such as excessive smartphone use, due to social desirability bias, or may inaccurately recall dietary habits and lifestyle activities, resulting in recall bias.

## 5 Limitations

This study presents several limitations that warrant consideration. The primary limitation pertains to the sampling strategy employed. Specifically, the use of convenience sampling from a single institution, Xuzhou Medical University, considerably restricts the external validity and generalizability of the findings. The student population at a medical university may possess distinct characteristics, including increased academic demands and higher health literacy, relative to students from other disciplines or institutions. Therefore, the observed prevalence of smartphone addiction and its associations with lifestyle and dietary behaviors may not be representative of the broader population of Chinese college students. To enhance sample representativeness and strengthen the generalizability of future findings, subsequent research should utilize random sampling methods across multiple universities and diverse academic disciplines. Additionally, the cross-sectional design of the current study limits the ability to infer causal relationships, as confounding variables may influence the observed associations. Longitudinal research designs are recommended to monitor the temporal dynamics and interactions among smartphone addiction, dietary behaviors, and health-promoting lifestyles throughout different stages of university students’ development. Finally, reliance on convenience sampling and self-report questionnaires may introduce biases, such as reporting and recall biases, which could compromise the accuracy and interpretation of the results.

## 6 Conclusion

This study examined the impact of smartphone addiction on eating behaviors and healthy lifestyle practices among students in Xuzhou, China. The findings indicate that smartphone addiction is positively correlated with higher intuitive eating scores, suggesting that individuals with greater addiction levels exhibit increased awareness of hunger and satiety cues. Conversely, smartphone addiction is negatively associated with healthy lifestyle scores, as it diminishes time allocated to physical exercise, social interactions, and adherence to regular routines. Dietary behavior serves as a mediating factor, linking smartphone addiction to overall healthy lifestyle outcomes. Specifically, the influence of addiction on eating habits indirectly undermines broader health-promoting behaviors. Furthermore, negative emotional states stemming from smartphone addiction are related to unhealthy eating patterns, which subsequently adversely affect physical health, exercise motivation, and social confidence, thereby impeding the maintenance of a healthy lifestyle.

## Data Availability

The original contributions presented in the study are included in the article/supplementary material. Further inquiries can be directed to the corresponding authors.
